# Cytoprotective effect of propolis on heat stress induces alteration to histological, ultrastructural, and oxidative stress in catfish (*Clarias gariepinus*)

**DOI:** 10.1007/s11356-023-30386-y

**Published:** 2023-10-19

**Authors:** Mariana S. Alfons, Ahmed Th.A. Ibrahim, Ahmed S. A. Harabawy, Mohamed B. Al-salahy, Gamal Badr

**Affiliations:** 1 Zoology Department, Faculty of Science, Fish Biology Lab, New Valley University, New Valley, El Kharga, 72511 Egypt; 2 Zoology Department, Faculty of Science, Assiut University, Assiut, 71516 Egypt; 3 Laboratory of Immunology, Zoology Department, Faculty of Science, Assiut University, Assiut, 71516 Egypt

**Keywords:** Heat stress, Immune response, Thymus, Spleen, Antioxidant, Propolis, Catfish

## Abstract

Our study helps to evaluate the immune response, antioxidative status, and resistance against heat stress (HS) in *Clarias gariepinus* treated with propolis extraction; the results will contribute to theories of fish physiology and immunity under high-temperature conditions. Forty-five fish were divided into three equal groups: the control, the HS group at 36 °C, and the HS treated with alcoholic extraction of propolis that dissolved in water for 3 weeks. The results of our study suggested that the stress response differs among tissues thymus, spleen, and liver. All the tissues showed alteration in morphological and cytological structure at the light microscope (LM) and transmission electron microscope (TEM); thymus showed edema and thymocyte destruction; the spleen detected collagen deposition, and the liver displayed endoplasmic reticulum amplification (ER). In addition, we examined oxidative stress and antioxidant defenses (lipid peroxidation, catalase, and glutathione) of the spleen and measured blood biochemical parameters (alanine transaminase and aspartic transaminase levels) after heat stress. However, this toxic effect of HS was neutralized by the propolis extraction. To conclude, propolis is recommended to cope with the impacts of heat stress on catfish (*Clarias gariepinus*) by improving immunity and antioxidative resistance.

## Introduction

Global climate change is a serious and growing threat to natural systems and their inhabitants. Temperature variations hurt aquatic ecosystems in particular because they are incapable of maintaining a steady body temperature and may thus be considered an ideal model for examining the influence of temperature (Cline et al*.* 2020). Increased temperature puts organisms under stress on numerous levels, including molecular, biochemical, physiological, and behavioral.

In fish, the same as in mammals, the innate immune system serves as the first line of defense, protecting them against hyperthermia stress, which has been proven to cause immunological damage and inflammatory reactions (Lu et al. 2016; Liu et al. 2019). The thymus and spleen are major lymphoid organs that exhibit dynamic physiological changes and are extremely susceptible to stress (Rauta et al. 2012).

The thymus is a critical tissue in the formation and maturation of T cells and in the development of the immune system and immunological responses (Bowden et al. 2005 and Cao et al. 2017). The spleen is the most important peripheral lymphoid organ, having a white pulp that stimulates hematopoiesis and the creation of defense cells, and a red pulp that triggers phagocytosis of old or faulty cells (Uribe et al. 2011). Distinct tissues may be more or less exposed to environmental change due to their variable functions and location within the body, resulting in different cellular stress responses purely due to temperature (Cui et al. 2013). The liver is the major target organ for heat stress since it is the primary metabolic and heat-producing organ. Several investigations have demonstrated that heat stress can cause liver damage (Agrawal and Gupta 2013).

Histological and cytological biomarkers are excellent risk assessment tools because cellular composition and/or structure changes reveal the net outcome of negative biochemical and physiological changes inside the cell. Direct monitoring of cellular modification allows for the discovery of sub-lethal effects in situ, which may serve as early warning signs of long-term damage caused by heat stress (Salazar-Lugo et al. 2011; Wang et al. 2018).

Thermal stress induces oxidative stress in organisms through the production of reactive oxygen species (ROS) and the organism’s inability to detoxify the ROS active species or repair injury (Zhao et al. 2017). To prevent oxidative stress and keep cellular redox state in balance, aerobic organisms have evolved efficient antioxidant defense systems, which include superoxide dismutase (SOD), catalase (CAT), and glutathione (GSH), as the antioxidant defense system is activated as a survival strategy to eliminate the extra ROS caused by heat stress (Madeira et al. 2016). To deal with these injuries, heat stress causes a diminution in liver function due to damaged hepatocytes and other cellular injuries, resulting in changes in alanine transaminase (ALT) and aspartic transaminase (AST) activity (Lu et al. 2016).

Several nutritional modifications, including dietary functional feed additive supplementation, have been used to help fish cope with the negative consequences of heat stress (Yonar et al. 2014; Hassaan et al. 2019). Propolis has piqued the interest of biologists because of its many biological activities and medicinal characteristics. Honeybees create propolis, a sticky, resinous substance used in the building and maintenance of their hives. It is prepared by combining the waxes produced by honeybees with resins gathered from various plants. Polyphenols (flavonoid, phenolic acids, and their esters, phenolic aldehydes), alcohols and ketones, quinones, coumarins, steroids, amino acids, and inorganic chemicals are all found in propolis (Araujo et al. 2012). Propolis has been recognized as a safe feed additive in many culture and livestock systems to improve the feed digest ability and gastric microbial community, enhance antioxidative and immune response as well as productive and reproductive performance, and reduce mortalities and histopathological alterations of body organs (Li and Kim 2014; Dotta et al. 2015). However, there is no information regarding the effects of propolis supplementation on catfish (*Clarias gariepinus*) under heat stress. As a result, we employed *C. gariepinus* to illustrate the immunotoxic impact of heat stress. Moreover, it can survive extreme environmental stress and has been utilized effectively in ecotoxicological research on a variety of xenobiotics (Kumari et al. 2016). We chose the fish because it has easily recognizable immunological organs, is scaleless, and is widely available all year.

In the present study, histology and cytology were analyzed to evaluate the comprehensive response of the thymus, spleen, and liver in catfish. The results of this study will benefit further investigations of the immune status of immune organs in a catfish under heat stress, as well as the influence of propolis as an anti-inflammatory and antioxidant by improving thymus, spleen, and liver architecture, cellularity, and function.

## Material and method

### Experimental animals and system design

#### Propolis

Propolis gained from Etman for honeybee products, Tanta, Egypt. It was cut into small pieces and taken 1.5 gm (50 mg/L) which dissolved in 10 mL of 70% ethanol then filtration and added 10 mL of solution to 30 L of water tank according to Talas and Gulhan (2009).

#### Experimental design

*Clarias gariepinus* was obtained from the Nile River. Before starting the experiment, fish were acclimatized to laboratory conditions for 3 weeks. Then, a total of 45 fish of similar size (250–300 gm) were randomly distributed into 9 (3 treatments × 3 replicates) glass tanks (80 cm × 35 cm × 40 cm) with 5 individuals per tank. Catfish were maintained at 28 °C (control group); catfish were gradually exposed to an experimental temperature of 36 °C (heat stress group) by thermostat heaters (REI-SEA, 300 W, Japan), and catfish were exposed to 36 °C supplemented with propolis (heat stress group + propolis) every day for 21 days at 2 h. This temperature was monitored by a thermometer during the heating period. During the experiment, catfish fed w3-4% body weight twice a day; removal of 5–6% water from the tanks was performed to remove leftover feed and excreta, and replenishment of the same volume and temperature of freshwater was provided in each group. The water was changed day after day to keep concentrations of propolis near the nominal level from the propolis stock solution. The skin and gills of catfish from the holding tank were free of parasites. The photoperiod was maintained at 16-h light and 8-h dark, and water quality parameters were monitored weekly to ensure that the conditions remained within ranges acceptable for fish growth; the dissolved oxygen concentration of the water was > 6 mg/L, and the pH remained between 7.60 and 7.80 throughout the study.

### Tissue sections for histological and histochemical study

At the end of the experimental period, fish were collected from each tank. Tissues thymus, spleen, and liver were dissected out from each fish and, after 24 h in formal alcohol fixative, were preserved in 70% ethanol. Samples were dehydrated in a graded series of ethanol, cleaned with methyl benzoate, and then embedded in paraffin wax. Sections of 5 µm were cut and stained with hematoxylin and eosin. Tissue sections were observed by a light microscope. For the histochemistry study, the tissue section was stained by Masson trichrome’s and Sirius red stains according to standard protocol.

### Tissue sections for transmission electron microscopy (TEM) analysis

Liver, spleen, and thymus samples were fixed in 2.5% glutaraldehyde in 0.1 M phosphate-buffered saline (PBS, pH 7.2–7.4), and the tissues were washed in 0.1 M PBS overnight. The tissues were fixed with 1% osmium tetroxide for 1 h at 4 °C, washed in 0.1 M PBS, and then dehydrated with graded ethyl alcohol and propylene oxide. Dehydrated tissues were embedded in araldite blocks. Ultrathin sections were obtained using an ultramicrotome and collected on copper grids for uranyl acetate staining. Stained sections were observed under a transmission electron microscope at the Electron Microscopic Center, Assiut University. Alteration cytology of tissues was monitored.

### Biochemical parameters

A blood sample was drawn from the caudal vein using heparinized syringes and transferred immediately into a test tube containing dried EDTA. Blood biochemical parameters as the activities of aspartate aminotransferase (AST) AS1061 and alanine aminotransferase (ALT) AL1031 were determined colorimetrically using assay kits (Spectrum Diagnostics, Egypt) according to the methods described by Reitman and Frankel’s (1957).

### Antioxidative and oxidative stress parameters

The animals were eviscerated for the removal of organs (spleen) from each group, flash frozen in liquid nitrogen, and then stored in the ultra‐freezer (− 80 °C). The organs were homogenized in 2 mL of phosphate buffered saline solution (0.14 M NaCl, 0.003 M KCl, 0.01 M Na2HPO4, 0.002 M KH2PO4, pH7.4, centrifuged (10,000 g, 20 min at 4 °C), and the pellets were discarded. The supernatants were employed to determine the antioxidant markers (superoxide dismutase (SOD), catalase (CAT) and glutathione (GSH), and oxidative stress (MDA levels). Antioxidant and oxidative stress markers were assayed according to the manufacturer’s protocol (MDA; CAT No.: MD 2529, CAT; CAT. No.: CA 2517; GSH; CAT. No.: GR 2511; Bio diagnostic, Dokki, Egypt).

### Statistical analysis

Analysis was carried out using Graph Pad Prism software version 5. Homogeneous data were evaluated using Tukey’s post-test. The level of significance was chosen at *P* < 0.05 between groups, and all the results were presented as mean ± SE (standard error of the mean).

## Result

### HS causes alteration of thymus catfish structure

Anatomically, the thymus gland of catfish is bilaterally placed and triangular and un-lobulated organ. This gland is located at the uppermost part of the mid-dorso-posterior part of the gill chamber behind the modified gills of catfish near to head kidney and is separated from the kidney by a bony structure.

**Fig. 1:**
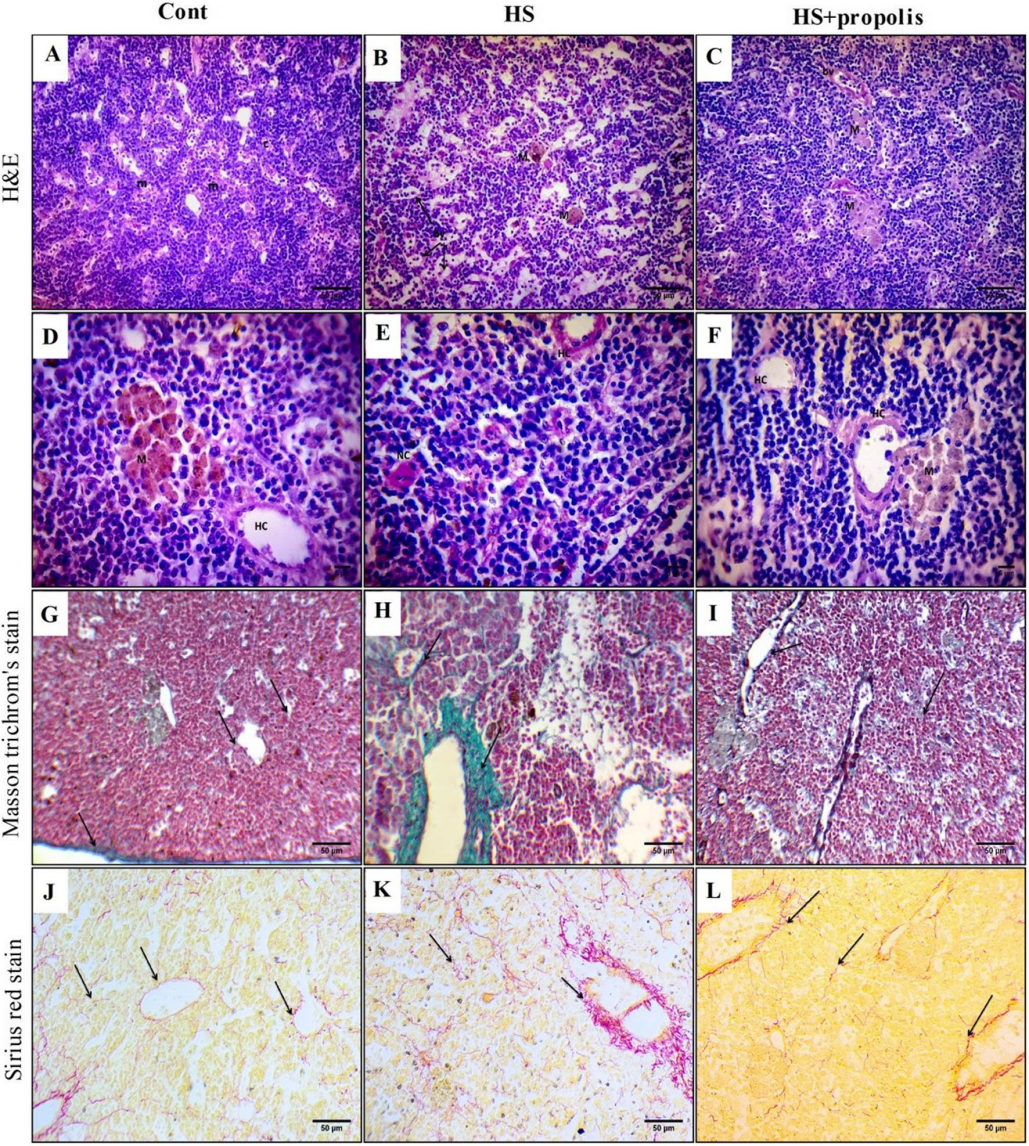
The effects of HS and propolis treatment on histology and histochemistry of structure of thymus. **A**, **D** Section of control catfish thymus showed to consist of the cortex (c) and medulla (m). Hassall’s corpuscle (HC) is formed of a concentric ring structure formed by multilayered epithelial cells and the presence of a melanomacrophage center (M) (H&E, × 400 and × 1000). **B**, **E** Section of heat stress groups of catfish thymus showed disappearance of thymic architecture. Edema (E) between cells and an increase in melanomacrophage centers (M) were noticed. Widening of lymphatic blood vessels and an increase in inflammatory cells were observed. Hassall’s corpuscle surrounded connective tissues and nurse cells were detected (H&E, × 400 and × 1000). **C**, **F** Section of heat stress catfish spleen treated with propolis showed upgrading of thymic cells and melanomacrophage cells (M). Multilayered Hassel corpuscles surrounded by deeply stained thymocytes were observed (H&E, × 400 and × 1000). **G** Section of control catfish thymus showed localization of collagen fibers in the capsule and around blood vessels (arrows) (Masson’s trichrome stain, × 400). **J** Section of control catfish thymus which stained collagen red color in localized in the underground of thymic cells and around blood vessels (arrows) (Sirius red stain, × 400). **H** Section of heat stress catfish thymus showed an increase of collagenous fiber mainly around blood vessels, faint network of thymus underground and in the capsule (arrows) (Masson trichrome’s stain, × 400). **K** Section of heat stress groups of catfish thymus showed red color as collagenous fiber mainly around blood vessels and network of thymus underground (Sirius red stain, × 400). **I** Section of heat stress catfish spleen treated with propolis showed diminished deposition of collagenous fiber mainly around blood vessels and the faint network of thymus underground (arrows) (Masson trichrome’s stain, × 400). **L** Section of heat stress catfish thymus treated with propolis showed diminished deposition of collagenous fiber mainly around blood vessels and a faint network of thymus underground (arrows) (Sirius red stain, × 400)

The thymus gland is composed of lymphocytes (thymocytes) which represent the most numerous cellular populations of the thymus. It is characterized by the cortex and medulla, and boundaries between the cortex and medulla are not sharply defined. A large number of lymphoid cells are frequently prominent within the thymic stroma of catfish. The thymic cortex is crowded with lymphoid cells with deeply stained nuclei and scant cytoplasm. These cells were smaller in volume and stained more heavily than cells in the medulla, and the density of cortex cells was higher than that in the medulla. Epithelial cells in the thymus gland form a network with their cytoplasmic processes that increase the area of interaction with thymocytes and support the movement of lymphoid cells. Hassall’s corpuscle (HC), formed of concentric ring structure formed by multilayered epithelial cells, and the presence of melanomacrophage center and cells are observed in Fig. 1A and D. Examination of the thymus section which exposure to HS exhibited alteration of the parenchymal structure resulted in the disappearance of thymic morphology. Newly formed thymocytes were implanted in connective tissues of the thymus gland between lymphoid follicles which appeared deeply stained. Large areas of the thymic parenchyma showed intercellular edema, where a marked decrease in the cellular density of the parenchyma occurred, and an increase in melanomacrophage centers was noticed. Widening of lymphatic blood vessels and increase of inflammatory cells were observed in Fig. 1B and E. The thymus tissue showed improvement after exposure to propolis when compared to HS more or less similar to control groups (Fig. 1C, E).

Masson trichrome’s and Sirrus red stains of the control group showed the distribution of collagenous fiber mainly in the capsule and a faint network of thymus underground and around blood vessels (Fig. 1G, J), but in the HS group showed increased deposition of collagenous fiber mainly around blood vessels, a faint network of thymus underground and in the capsule (Fig. 1H, K); however, in the HS group, supplementation with propolis exposure reduces the red color of thymic cells in the underground and around blood vessels (Fig. 1I, L).

### The effect of propolis supplementation on the structure of thymocytes and improved immune response during HS

**Fig. 2:**
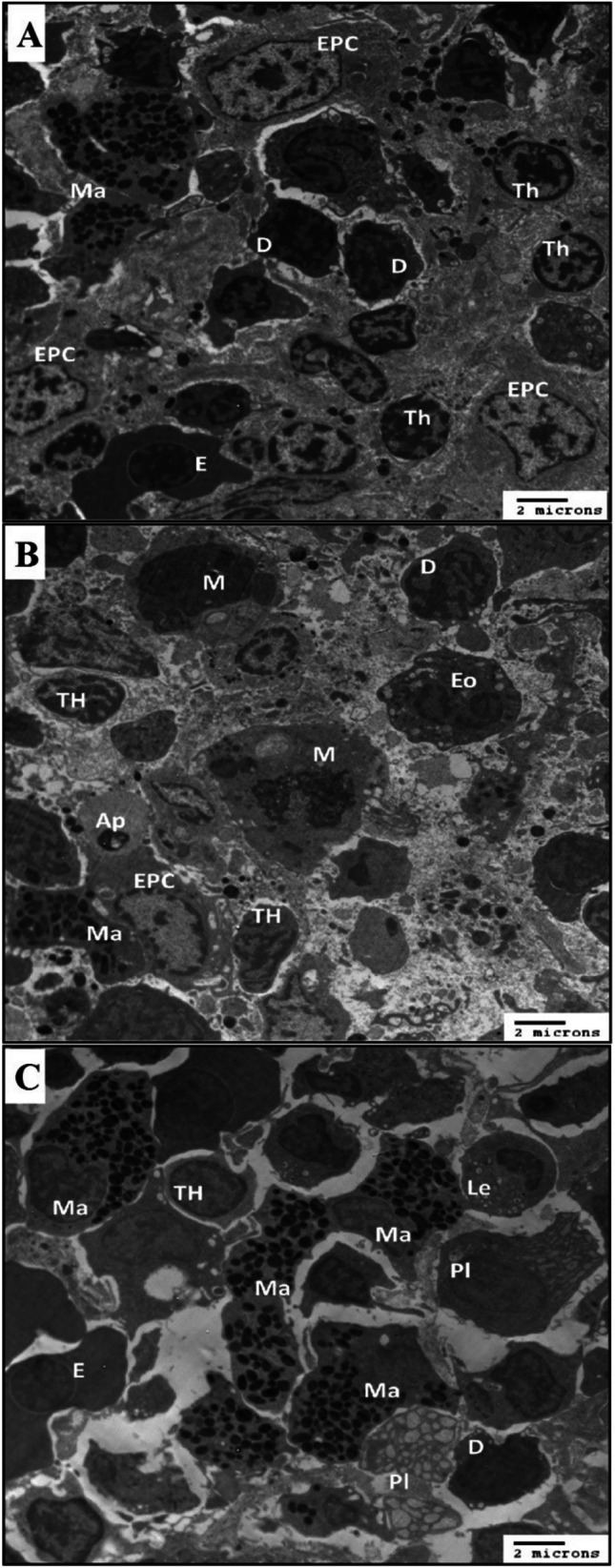
The effects of propolis treatment on the ultrastructure of the thymus. **A** Electron micrograph thymus of control catfish showed erythrocyte (E). Thymocytes (TH). The dendritic cells showed pseudopodia, an indented heterochromatic nucleus, and many cytoplasmic vacuoles (D). Reticular epithelial cells are characterized by open-phase nuclei with many cytoplasmic vacuoles (REC). Mast cells are characterized by electron-dense granules (Ma). **B** Electron micrograph thymus of HS catfish showed degenerated thymocytes (TH). Macrophage has processes of pseudopodia, and their cytoplasm was occupied with phagosomes (M). The dendritic cell showed pseudopodia, an indented heterochromatic nucleus, and many cytoplasmic vacuoles (D). Reticular epithelial cells (REC) are characterized by open-phase nuclei with many cytoplasmic vacuoles. Mast cells are characterized by electron-dense granules (Ma). Apoptotic cell (AP). **C** An electron micrograph of the thymus of HS catfish supplementation with propolis showed thymocytes (TH). Erythrocyte (E). Mast cells are characterized by electron-dense granules (Ma). Plasma cell (Pl) is characterized by many rough endoplasmic reticulum. Immature leukocyte (Le)

TEM of the control thymus of catfish showed a population of thymocytes of different sizes. Thymocytes contain thin pale cytoplasm and rounded nuclei with thin, dark peripheral heterochromatin and dense aggregation of islands. Reticular epithelial cells had large elongated or rounded bodies containing heterogeneous electron-lucent cytoplasm with organelles and few processes. Irregular and open-phase nuclei with prominent nucleoli were observed. Few mast cells, also known as the mastocyte or labrocyte, contain pale cytoplasm with different sizes and shapes of electron-dense granules. The dendritic cell showed few pseudopodia with slightly pale cytoplasm tiny vacuoles and large irregular nuclei containing dense chromatin (Fig. 2A). Heat stress–induced histopathological changes in all thymic tissues and increased connectives tissues matrix and debris between cells were observed; thymocytes contain light cytoplasm, and large nuclei with dark heterochromatin aggregated in both periphery and islands other thymic cells were degenerated or apoptotic containing part of cytoplasm and degenerated nuclei. Parts of macrophages contain slightly dark cytoplasm with many phagosomes’ electron electron-dense bodies. Dendritic cells showed the same morphology as the control groups. Reticular epithelial cells appeared as large and elongated bodies containing heterogeneous electron-lucent cytoplasm with vacuoles and processes. A slightly irregular and heterochromatic nucleus was seen. Part of the mast cell was noticed to contain electron-dense of different sizes and shape granules. Some cells become apoptotic bodies (Fig. 2B).

Heat stress supplementation with propolis showed amelioration in the morphology of thymocytes which appeared with slightly irregular boundaries and contained a ring of slightly dense cytoplasm; rounded open-phase nuclei were observed. Mast cells were increased in number, which takes irregular different shapes containing slightly dense cytoplasm with different sizes and shapes of electron-dense granules. Euchromatic and irregular shape nuclei are located peripherally. Plasma cells and many parts of other cells were seen with irregular large bodies containing slightly dense cytoplasm full of short cisternae of the rough endoplasmic reticulum. Oval shape euchromatic nucleus slightly eccentric in position was noticed. Dendritic cells showed few pseudopodia with slightly electron-dense cytoplasm and tiny peripheral vacuoles. It was seen as a large irregular euchromatic nucleus as compared with HS groups. Reticular epithelial cells had large bodies with pseudopodia and contained heterogeneous slightly electron-dense cytoplasm, vacuoles, and phagosomes. Oval shape euchromatic nucleus with prominent nucleoli is observed in Fig. 2C.

### HS induces collagen fiber in the catfish spleen

**Fig. 3:**
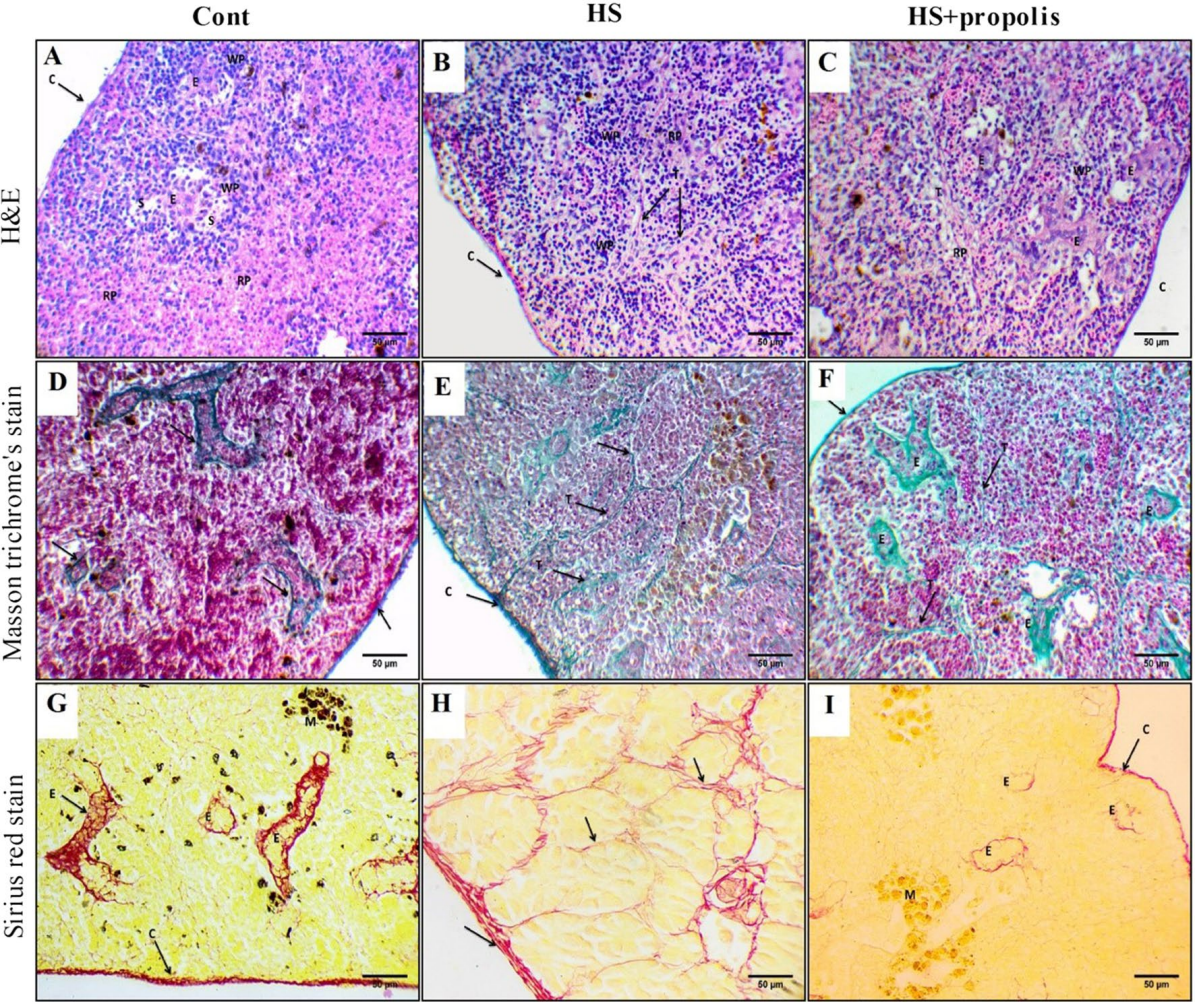
Effect of HS and propolis supplementation on the spleen architecture. **A** Section of control catfish spleen covered by thin connective tissue capsule (c) which ramified into fine trabecula aggregation of lymphocytes represents white pulp (WP). The main structure is red pulp (RP) which contains blood sinus (S) full of blood cells contained in their core ellipsoid structure (E). Few melanomacrophage (M) cells were noticed localized in the red pulp (H&E, × 400). **B** Section of heat stress groups of catfish spleen showed marked lobules surrounded by thick trabecula (T) stained acidophilic. Thickening (TH) of acidophilic connective around blood vessels which were engorged by blood cells and slight hemorrhage were noticed. Increase of melanomacrophages centers (M) which contain brown hemosiderin pigments. Increase of aggregated lymphocytes in white pulp (WP). Red pulp (RP) underground stained acidophilic contains few lymphocytes and blood capillaries. Increase of both lymphocytes (L) and melanomacrophages centers (M) which contain brown hemosiderin pigments (H&E, × 400). **C** Section of heat stress catfish spleen treated with propolis covered by thin connective tissue capsule (c) which ramified into fine trabecular. Few aggregations of lymphocytes represent white pulp (WP). The dominant structure is red pulp (RP) which contains congested blood capillaries and an ellipsoid structure (E). An increase in different shapes of ellipsoid structures was noted (E). Decrease the lobulation of connective tissue fibers when compared with (HS) groups (H&E, × 400). **D** Section of control catfish spleen showed localization of collagen fibers in capsule (C) and connective tissues surrounding the ellipsoid structures (E) which appeared with irregular different shapes (arrow) (Masson’s trichrome stain, × 400). **G** Section of control catfish spleen which stained collagen red color in localized in capsule (C) and underground of ellipsoid structures (E) (Sirius red stain, × 400). **E** Section of heat stress of spleen catfish showed localization of dense and thick collagen fibers in capsule (C), surrounded blood vessels (bv), around the splenic lobulation and ellipsoid structures (E) (Masson trichrome’s stain, × 400). **H** Section of heat stress of spleen showed a broad distribution of fibrotic collagen around splenic lobules (T) and of ellipsoid structures (E). Section of heat stress of spleen catfish showed a broad distribution of fibrotic collagen in red color around blood vessels (bv) (Sirius red stain, × 400). **F** Section of heat stress catfish spleen treated with propolis showed localization of thin collagen fibers in capsule (C), around lobulation (T), and slightly dense around ellipsoid structure ground substances (E) (Masson trichrome’s stain, × 400). **I** Section of heat stress catfish spleen treated with propolis showed a decrease in staining of fibrotic collagen in capsule, ellipsoid structures underground (E), and faint reaction around splenic lobules (Sirius red stain, × 400)

The spleen is covered by a thin, fibrous capsule with little evidence of contractile ability. Fine trabeculae were present. The main dominant splenic structures are red pulp, an interconnecting system of splenic cords and sinusoid capillaries (open capillaries); white pulp in catfish is poorly developed and consists mainly of diffusely distributed lymphoid cells with a defined marginal zone present. White pulp contains s groups of blood sinuses, and ellipsoid structures located in their core and melanomacrophage cells (MMCs) were also present. Ellipsoid structures are present in both red and white pulps which are terminal capillaries showing a thin endothelial cell layer consisting of cubic cells with centrally located rounded nuclei surrounded by a sheath of acidophilic fibrous connective tissues and a chain of aggregated cells lymphocytes and macrophages (Fig. 3A). Examination of the spleen section which exposure to HS exhibited an increase in white pulp size contains newly formed lymphocyte which surrounded by thick trabecular of acidophilic connective tissues, and vascular congestion and hemorrhage were evident. Expansion of the ellipsoid structures which were surrounded by thick sheaths of acidophilic connective tissues was observed. The shrunken red pulp contains dispersed blood cells, and melanomacrophage centers with large, irregular, brown granular pigments (likely hemosiderin), fibroblastic proliferation, and dilated sinusoids are noticed in Fig. 3B. More improvements were observed in heat stress treated by propolis. However, the red pulp contains drainage of blood cells, congestion of blood capillaries, and marked ellipsoid structure with thick sheath are noticed in Fig. 3C.

Masson trichrome’s stain exposed a thin amount of collagen fibers in the capsule and trabeculae and surrounded the ellipsoid structures which appeared with irregular different shapes (Fig. 3D). Sirius red stain revealed collagen fiber, which was localized in the capsule, a fine network in underground substances, and concentrated around ellipsoid structures (Fig. 3G). Examination of the spleen of the HS catfish section by Masson trichrome’s stain showed an increase in dense and thick collagen fibers surrounding blood vessels, around the splenic lobulation, and in ellipsoid structures. Also, dense collagen fibers in the capsule and around the splenic lobulation are seen in Fig. 3E. Examination of the spleen of the HS catfish section by Sirius red stain indicated a broad distribution of fibrotic collagen around splenic lobules, splenic matrix, and ellipsoid structures (Fig. 3H). Examination of spleen of HS treatment with the propolis catfish section by Masson trichrome’s stain showed localization of thin collagen fibers in capsule, around lobulation and slightly dense around ellipsoid structure ground substances (Fig. 3F). Examination of spleen of HS treatment with the propolis catfish section by Sirius red stain showed a decrease in staining of fibrotic collagen in capsule and ellipsoid structures underground and a faint reaction around splenic lobules (Fig. 3I).

### Propolis supplementation during HS reduces damage in spleen catfish

**Fig. 4:**
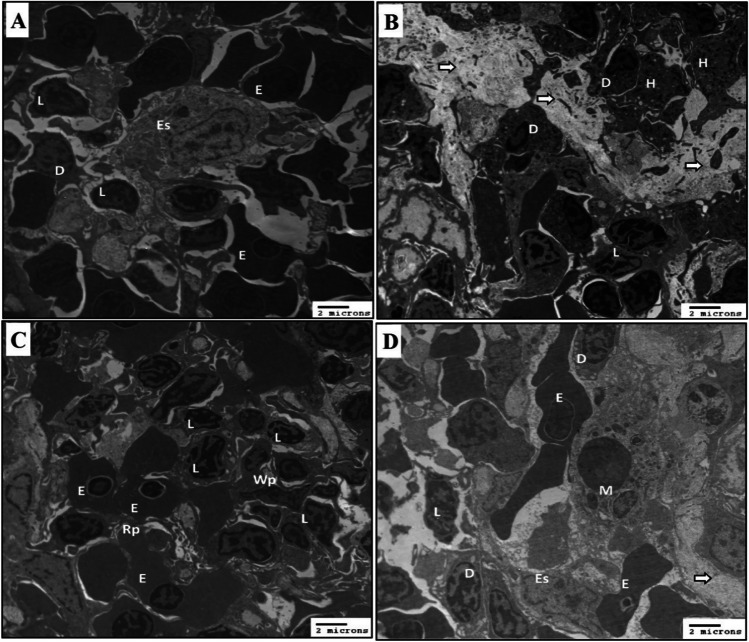
Effect of HS and propolis supplementation on the ultrastructure of spleen. **A** Electron micrograph of spleen of control catfish showed erythrocyte (E). Lymphocytes showed an irregular nucleus (L). Dendritic cells showed pseudopodia, indented heterochromatic nucleus, and cytoplasmic vacuoles (D). Lining cell of ellipsoid (Es). **B** Electron micrograph of the spleen of HS catfish showed lymphocytes showed an irregular nucleus (L). Dendritic cells showed pseudopodia, indented heterochromatic nucleus, and cytoplasmic vacuoles (D). Heterophils are characterized by many vacuoles and rod-like granules (H). Collagen fiber (arrows). **C** Electron micrograph of the spleen of HS catfish showed red pulp (Rp) containing erythrocytes (E) and white pulp (Wp) containing lymphocytes (L). **D** Electron micrograph of spleen of HS catfish supplementation with propolis showed erythrocytes (E). Lymphocytes showed an irregular nucleus (L). Dendritic cells showed pseudopodia, indented heterochromatic nucleus, and cytoplasmic vacuoles (D). Macrophage has processes of pseudopodia, and their cytoplasm was occupied with lysosomes and other particles (M). Collagen fiber (arrows). Lining cell of ellipsoid (Es)

Examination of the spleen of control catfish under TEM showed red and white pulps. The red pulp includes mature and degenerating erythrocytes. Mature erythrocytes with an irregular shape contain electron-dense cytoplasm with dark heterochromatic nuclei. White pulp contains different types of leucocytes, lymphocytes, and dendritic cells. Lymphocytes took a different shape from round to ovoid with slightly electron-dense cytoplasm with a few tiny vacuoles. Large heterochromatic nucleus occupied the whole body of the cell. Dendritic cells showed pseudopodia, with light-staining homogenous cytoplasm containing tiny vacuoles and open-phase euchromatic nucleus, with thin peripheral chromatin, and with aggregated dense chromatin islands, and telocyte cells were observed. The ellipsoid’s structure is the termination of blood sinus which is invested in regions of collagen fibers lined by cubic cells with processes containing electron-lucent cytoplasm and mitochondria and a dense body. Interphase slightly eccentric nucleus contains a thin dense nuclear envelope and aggregated chromatin islands (Fig. 4A).

Examination of the spleen of HS catfish under TEM showed collagen fibers which appear as grey-white staining deposited between splenic cells. Many degenerated splenocytes with multiple cytoplasmic vacuoles. Dendritic cells with irregular cell bodies contain electron-dense cytoplasm with tiny vacuoles and small dense granules, and its heterochromatic nucleus with peripheral condensed chromatin was seen. Heterophils appear as irregular cells with electron-dense cytoplasm large number of rod shapes and granules of different intensity (Fig. 4B). Sign of degeneration of irregularly distributed splenic cells such as aggregation of different size and shapes of lymphocytes contains slightly dense cytoplasm with condensation, and peripheral deposition of heterochromatin and condensed islands were observed. Deformation of erythrocyte shapes with slightly dense cytoplasm and a clear sign of nuclear degeneration such as nuclei shrunken was surrounded by a hallow space (Fig. 4C). Electron micrograph of spleen of HS catfish supplementation with propolis showed improvement in splenic cells irregular rim of erythrocytes with electron-dense cytoplasm with dark heterochromatic nuclei more or less similar to control groups. Part of the ellipsoids structure is surrounded by fewer amounts of collagen fibers when compared with HS groups. It is lined by irregular cubic cells and contains electron-lucent cytoplasm. An euchromatic nucleus with a thin dense nuclear envelope and few aggregated chromatin islands was noticed. Different-shaped lymphocytes with slightly irregular rims contain slightly electron-dense cytoplasm. Large heterochromatic nuclei occupy the whole body of the cell. Irregularity in dendritic cell boundary with slightly electron-dense heterogeneous cytoplasm which contains few tiny vacuoles was observed. Macrophage cell open-phase euchromatic, folded, and eccentric nucleus with electron-lucent cytoplasm which contain many electrons dense bodies of different size and autolysosome which contains debris was seen (Fig. 4D).

### Heat stress enhances oxidative stress and alters antioxidants

**Fig. 5:**
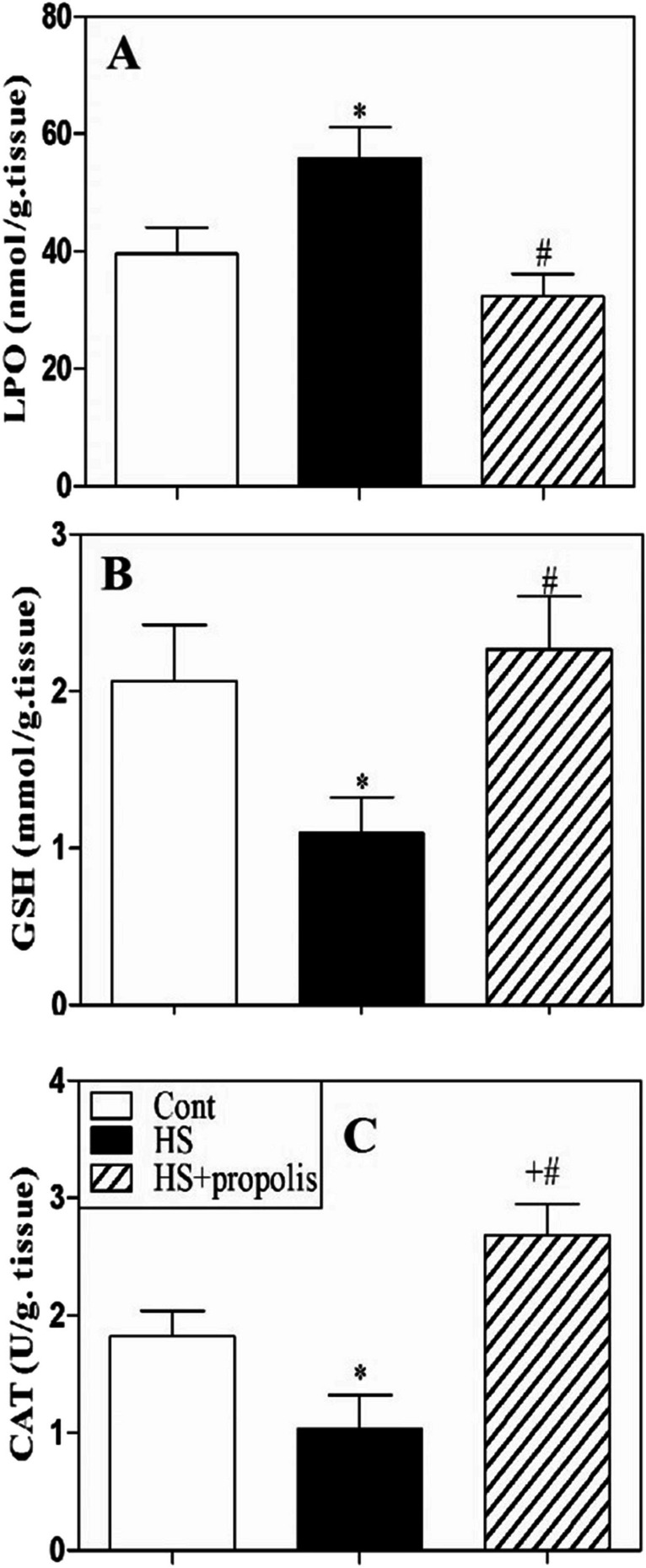
Propolis acts as an antioxidant in the spleen during exposure to HS. Oxidative stress markers such as lipid peroxidation LPO were monitored (**A**). The levels of glutathione (GSH) and catalase (CAT) (**B**, **C**) which accumulated data from three catfish from each group are expressed as the mean ± SEM (*n* = 5). ^*^P < 0.05 for HS catfish compared with control catfish, ^+^*P* < 0.05 for HS + propolis catfish compared with control catfish and.^#^*P* < 0.05 for HS + propolis catfish compared with HS catfish (ANOVA with Tukey’s post-test)

We then measured oxidative stress as lipid peroxidation (LPO) and the activities of some antioxidant enzymes (GSH and CAT) in the tissue lysates of the spleen. Accumulated data from five individual catfish from each group are expressed as the mean value ± SEM (Fig. 5). The results demonstrated that HS catfish exhibited significant (*P* < 0.05) elevations in lipid peroxidation marker in tissue lysates from the spleen. However, treatment of HS catfish with propolis supplementation significantly (*P* < 0.05) restored lipid peroxidation as the control catfish (Fig. 5A). Then, we measured the levels of GSH and the activities of some antioxidant enzymes as catalase in tissue lysates (expressed as U/g. tissue) exhibited significant (*P* < 0.05) downregulation in the HS catfish as compared to control catfish (Fig. 5B, C). Nonetheless, treatment of catfish with propolis significantly increases GSH and CAT.

### HS causes alteration of hepatocytes

**Fig. 6:**
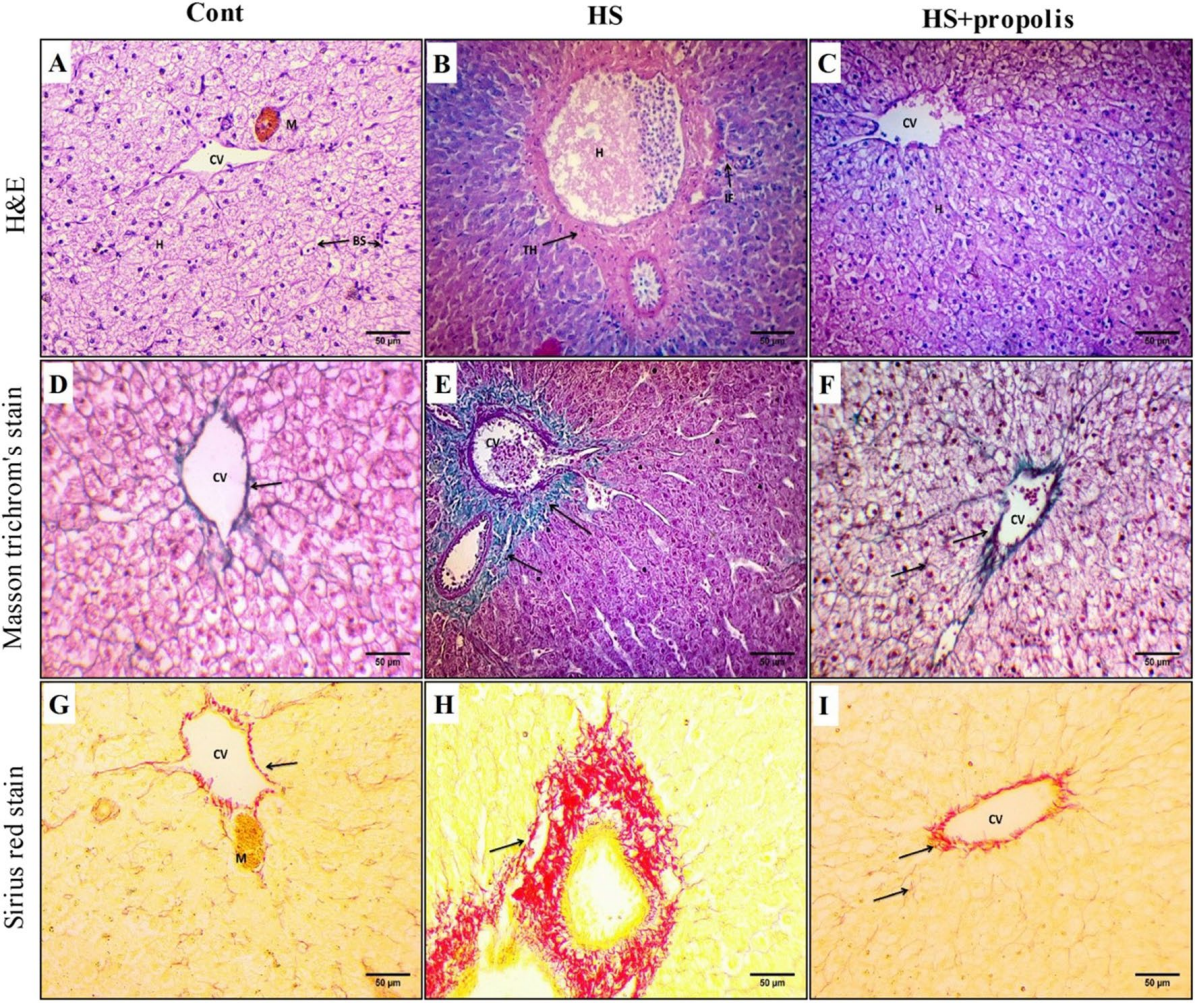
Propolis improves liver structure during HS. **A** Section of the fish liver of control groups appeared as a continuous mass of hepatic cells (H) around the central vein (CV) and interrupted by blood sinusoids (BS) (H&E, × 400). **B** Section of fish liver of heat stress showed thickening (TH) of connective tissues surrounding the portal area with congested and hemorrhage (H) in the portal vein. The hepatocytes have homogenous basophilic cytoplasm. An increase in inflammatory cells (IF) was noticed (H&E, × 400). **C** Section of fish liver of heat stress treated with propolis showed restoration of normal appearance hepatic strands around the central vein. Slight vacuolation was observed in some hepatocytes (H) (H&E, × 400). **D** Section of fish liver of control groups showed faint reaction for collage nous fiber localized around the central vein (arrows) (Masson trichrome’s stain, × 400). **E** Section of fish liver of heat stress of other groups showed an increase of collagenous fibers in the ground substance of portal area and around blood vessels (Masson trichrome’s stain, × 400). **F** Section of fish liver heat stress treated with propolis showed a decrease in collagenous fiber localized around the central vein (arrow) (Masson trichrome’s stain, × 400). **G** Section of the control group’s fish liver showed collagen deposition around the central vein and fine network between hepatocytes (arrow) (Sirius red stain, × 400). **H** Section of fish liver of heat stress groups showed an increase of collagenous fibers in the ground substance of portal area, around blood vessels, and between hepatocytes in blood sinusoids (arrow) (Sirius red stain, × 400). **I** Section of fish liver heat stress treated with propolis showed a decrease in collagenous fiber localized around the central vein (Sirius red stain, × 400)

Section of the liver of control groups exhibited normal histological structure of the parenchyma with large polygonal hepatocytes aligned in cords and separated by blood sinusoids and extended radially from the central veins to the periphery of the lobules till reached the portal area. Each hepatocyte contains fine granular acidophilic cytoplasm with a distinct vesicular, round, and centrally located nucleus with nucleolus. Blood sinusoids lined by endothelial cells are observed in Fig. 6A. The liver of the heat stress group exhibited disruption of hepatic tissue organization with thickening of connective tissues surrounding the portal area with congestion and hemorrhage in the portal vein. The hepatocytes have homogenous basophilic cytoplasm. An increase of inflammatory cells (IF) is noticed in Fig. 6B. HS catfish supplemented with propolis hepatic structures retained their normal appearance; polygonal hepatocyte with homogenous acidophilic cytoplasm and vesicular and centrally located nuclei. Decrease of melanomacrophage cells is presented in Fig. 6C.

Examination of liver catfish of the control group with Masson trichrome’s stain revealed a small number of connective tissue fibers (collagenous) around the central vein (Fig. 6D), and examination of liver catfish with Sirius red stain revealed a small number of connective tissue fibers around the blood vessels and fine fibers in connective tissues around blood sinusoids (Fig. 6G). The investigation of liver sections of the heat stress group stained with Masson’s trichrome stain showed a great accumulation of connective tissue fibers around the central vein, portal area, and in-ground substances that border blood sinusoids (Fig. 6E), and with Sirrus stain for fibrotic collagen showed, increased deposition of connective tissue fibers around the portal area and the central vein, and faintly dispersed fibers outline the blood sinusoids (Fig. 6H). Examination of liver sections of the propolis supplementation group stained with Masson’s trichrome and with Sirrus stain showed depletion of collagenous fibers around central veins, and few faint staining fibers between hepatocytes were observed when compared with HS groups more or less similar to control groups (Fig. 6F, I).

### Heat stress causes alteration of hepatocytes

**Fig. 7:**
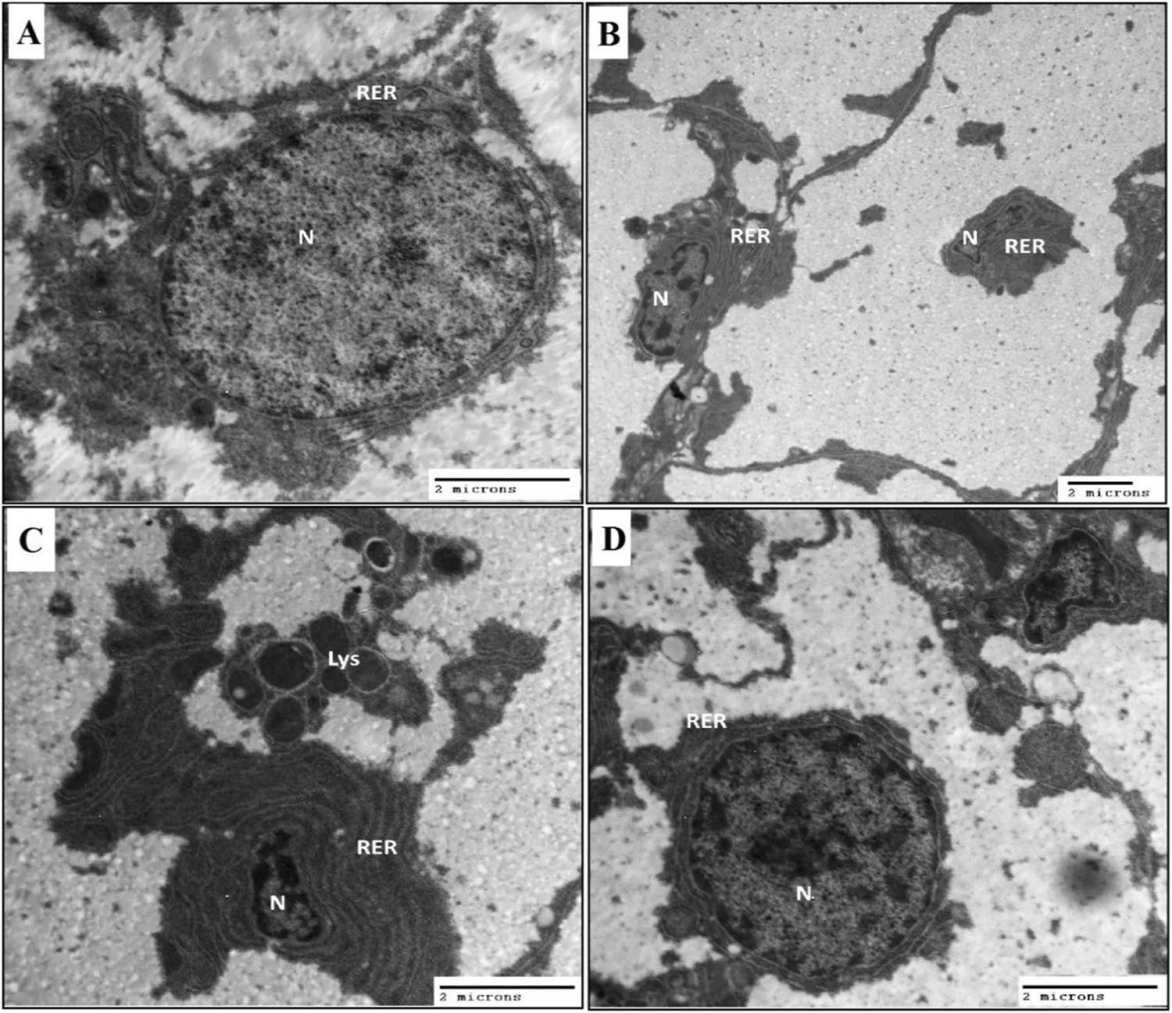
Effect of propolis supplementation on improvement of hepatocytes under heat stress. **A** Electron micrograph liver of control catfish showed nucleus of hepatocyte (N). Rough endoplasmic reticulum (RER). **B** Electron micrograph liver of HS catfish showed shrinkage nucleus of hepatocyte (N). Rough endoplasmic reticulum (RER). **C** Electron micrograph liver of HS catfish showed shrinkage nucleus of hepatocyte (N). Rough endoplasmic reticulum (RER). Lysosomes (Lys). **D** Electron micrograph liver of HS catfish supplementation with propolis showed improved nucleus of hepatocyte (N). Rough endoplasmic reticulum (RER)

We next investigated the ultrastructure of hepatocytes during heat stress and treatment with propolis. Hepatocytes of control catfish revealed a centrally located and rounded nucleus that contains euchromatin and few granulated electron-dense chromatin islands. Electron-lucent cytoplasm was observed as rarified cytoplasm in which all cytoplasmic organelles aggregated perinucleus which surrounded by RER in concentric layers cisternae of Golgi bodies, and electron-dense vesicles were observed (Fig. 7A). While hepatocytes of HS groups showed a deformed shape of nucleus which appeared eccentrically located and shrunken with condensed and aggregated heterochromatin at periphery, few euchromatins were noticed as rarified cytoplasm in which all cytoplasmic organelles aggregated pronucleus. An increase in the concentric layer of RER with dilatation or vacuolation and secondary lysosomes is observed in Fig. 7B and C. Amelioration hepatocytes of heat stress supplementation with propolis which appeared more or less control groups. Centrally located and rounded nucleus contains euchromatin and a few granulated electron-dense chromatin islands. Electron-lucent cytoplasm was observed as rarified cytoplasm in which all cytoplasmic organelles aggregated perinuclus which surrounded by RER in concentric layers. Cisternae of Golgi bodies and electron-dense vesicles are observed in Fig. 7D.

### Heat stress regulates liver function

**Fig. 8:**
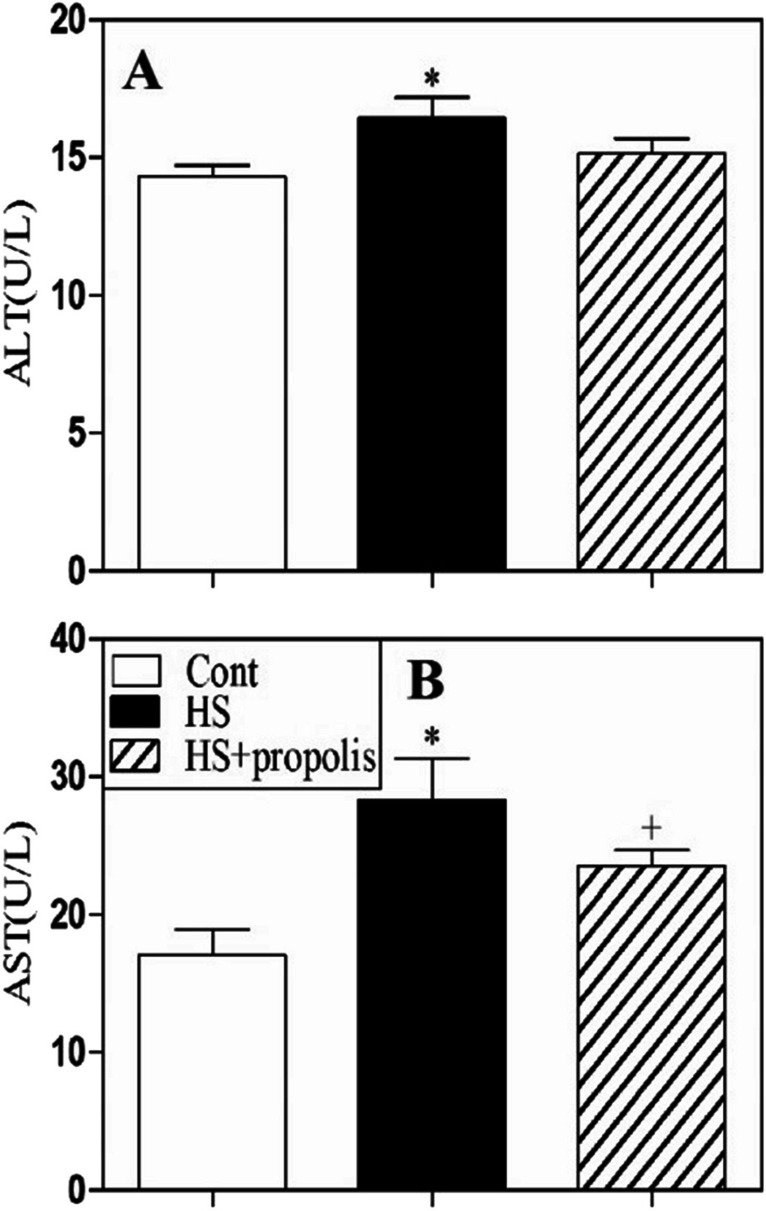
The effects of HS and propolis on biochemical parameters. Blood samples were collected; the activities of ALT (**A**) and AST (**B**) were measured. The results from 5 catfish from each group are expressed as the mean level of each parameter ± SEM. ^*^P < 0.05 for HS versus control catfish. ^#^*P* < 0.05 for HS with propolis catfish vs. HS catfish. ^+^*P* < 0.05 for HS + propolis catfish versus control catfish (ANOVA with Tukey’s post-test)

Blood samples were collected from five catfish from each group’s control, HS, and HS supplementation with propolis. The activities of aspartate aminotransferase (AST) and alanine aminotransferase (ALT) were measured. The results exhibited significant elevations in (AST) and (ALT) activities in serum in response to HS exposure in catfish in comparison with corresponding values of negative controls. Propolis supplementation induced either relative or complete improvements of AST and ALT activities, respectively, in the group compared with corresponding negative control values (Fig. 8A, B).

## Discussion

Extreme temperature occurrences caused by climate change have posed substantial and growing problems to aqua farmers and farmed fish during the last three decades. Fish physiology, metabolism, growth, and productivity are all affected by extreme heat occurrences. Heat stress has multiple impacts, causing obvious damage on their morphological structures. In the present study, the improved immune response of fish fed a diet supplemented with propolis extract may be due to the beneficial effect of propolis on the anti-allergic and anti-inflammatory activity of the propolis extract components.

The most serious damage was observed in immune organs such as the thymus and spleen under HS. The thymus gland is the primary center of development and differentiation of T-lymphocytes, playing an important role in cellular and humoral immunities so it is very sensitive to stress (Cao et al. 2017). In the present study, obvious alterations in thymus structure were observed in the HS group, which are cellular damage of thymocytes and cell debris. The variations in thymus histopathology and architecture are of particular relevance for the determination of immunotoxicity (Kuper et al. 2000). HS catfish groups with propolis treatment expose enhancement cells of the thymus gland and also the influx of inflammatory cells into the thymus. Propolis may stimulate greater antibody production (plasma cells), and it acts as an anti-inflammatory (mast cells). These immunomodulatory properties of propolis are contributed by its flavonoids and phenolic acids. Recently, it has been pointed out that dietary change might contribute to the onset of allergic diseases.

Also, the histopathological and ultrastructural alterations were observed in the spleen samples under increased temperature, and the splenocyte cells degenerated displayed extensive chromatin condensation and augmented collagen deposition. However, the HS group supplemented with propolis diet exposure increased enhancement similar to the control group. The finding of our studies suggests that propolis modulates non-specific immunity via the improvement of erythro-phagocytic activity and the improvement of the lymphocyte population in the white pulp in this tissue. The elevation of water temperature provokes physiological stress associated with excessive ROS generation which can attack the phospholipid membrane, resulting in mitochondrial dysfunction and ultimately affecting the normal energy metabolism of the spleen (Acar 2018). In the current study, catfish exposed to higher temperatures had significantly higher levels of lipid peroxidation than controls in spleen tissue, which was consistent with (Cui et al. 2013; Lu et al. 2016). To defend against the harmful effects of ROS, cells have developed numerous mechanisms as antioxidant defense systems which play an important role in against oxidative stress damage caused by excessive levels of oxygen-free radicals which are crucial for innate immunity (Dalvi et al. 2017). The antioxidant enzymes, such as CAT and GSH, might orchestrate the cellular defense against stress as key components of antioxidant defense (Almroth et al. 2015; Zhou et al. 2019). In the present data, the diminution of the activities of antioxidant enzymes CAT and GSH was observed in the spleen of *C. gariepinus* in the thermal stress group. The deficiency of increases in antioxidant enzymatic activity might explain why the observed increase of oxidative damage products in catfish defenses was insufficient to prevent damage. In the HS groups, supplementation with a propolis diet showed an elevation of antioxidant activities. The antioxidant activity of propolis and its constituents has been well documented (Osés et al. 2020), with the vast majority of outcomes demonstrating a reduction in oxidative stress markers (Aldemir et al. 2014). To reduce the oxidative stress (LPO) that induces tissue damage, endogenous antioxidant systems have developed protective mechanisms including enzymes, GSH, and CAT. In general, the immunological and antioxidant activities observed in the present research recommend that propolis can alleviate the negative effects of HS in catfish.

The liver carries out vital physiological functions, such as metabolism, excretion, and detoxification, and its state in an organism can best reflect the nutritional physiology and pathological state of the body (Sun et al. 2019). Many studies have shown that various environmental stresses can cause changes in liver structure and even affect its function (Qu et al. 2014; Qi et al. 2021). Histological and ultrastructural alterations were observed in the liver under thermal stress, and the hepatic cells were disintegrated, and irregular blood vessels with hemorrhage and infiltrated inflammatory cells were revealed in the liver. Ultrastructural analysis demonstrated that heat stress caused alterations of the hepatocyte’s ultrastructure, degeneration of hepatic organelles, and a remarkable proliferation of rough endoplasmic reticulum. This alteration causes reticuloendothelial system weakening and antigen-based immune protection. Also, a decrease in blood cell mobilization activity in extreme situations and extensive lipofilization of the liver prevents normal glycogenesis as well as normal metabolism levels (Fishelson 2006). Our study revealed that supplementation of propolis results in a protective effect on hepatocytes during thermal stress. These results agree with other readings that prove propolis incorporated into fish diets resulted in enhanced metabolism, improved immune response, and physiological performance in other fish species (Yonar et al. 2014; Acar 2018; Hassaan et al. 2019).

Monitoring the biochemical changes in hepatic enzyme activities such as ALT and AST is an indication of liver function (Hassaan et al. 2019). When liver damage or organ dysfunction occurs, their contents in the blood will change dramatically, so it is often used as a diagnostic indicator of liver damage (Atli et al. 2015). Meanwhile, this study found that the increased AST and ALT activities at higher temperatures suggest the mobilization of free amino acids for energy production. Similar observations have been reported in *C. carpio* (Ahmad et al. 2011) in response to thermal acclimation. Also, the elevation of liver enzyme activities may indicate enzyme leakage across damaged plasma membranes and/or increased synthesis of liver enzymes by the action of stress (Chen et al. 2017). In the present study, reduction in ALT and AST in catfish treated with aquas propolis extraction demonstrated that the components of propolis (flavonoids) have a hepatoprotective role. This agrees with Talas and Gulhan (2009), who reported that propolis has a protective effect on liver cells and their enzymes in rainbow trout.

The current investigation found that propolis was crucial in modulating and regulating both innate and adaptive immunity in *C. gariepinus.*

## Conclusions

In conclusion, the present study shows the importance of multi-organ approaches to comprehend the full health status of the organism’s response to increasing temperature. HS causes weakening of the catfish’s immune defense system, mild damage to the liver, loss of function of immune organs, and elevation of oxidative stress. Propolis would be an appropriate approach for safeguarding catfish from heat stress because it is an antioxidant, anti-inflammatory, and anti-allergy agent.

## Data Availability

The support data of the findings of this study are available from New Valley University, but limits apply to the availability of these data, which were used under license for the current study. Data are however available from the author upon reasonable request and with permission of New Valley University.
